# Teachers’ Perspectives on Social Media Networking for Indonesian Language Learning in Inclusive Elementary Schools: A Multi-Method Case Study

**DOI:** 10.12688/f1000research.172079.1

**Published:** 2025-11-24

**Authors:** Megan Asri Humaira, Rusi Rusmiati Aliyyah, Mega Febriani Sya, Rasmitadila

**Affiliations:** 1 Universitas Djuanda, Bogor, West Java, Indonesia

**Keywords:** inclusive school, national language, social media approach, special education, teacher perceptions

## Abstract

**Background:**

Effective learning strategies are essential for inclusive elementary classrooms, particularly in Indonesian language education, where students have diverse learning needs and digital access levels. Although social media has become a common educational tool, its structured use as a pedagogical model for inclusive learning in Indonesian schools is limited. Previous studies have largely focused on general online learning, leaving a gap in understanding how social media can foster inclusion and language skill development. This study addresses that gap by exploring how social media can be systematically integrated into Indonesian language learning and by developing a strategy that promotes inclusivity, collaboration, and student engagement.

**Methods:**

A qualitative case study approach was employed to capture in-depth insights from classroom practices. Data were collected from 15 elementary school teachers across five Indonesian provinces (West Java, Central Java, East Java, Yogyakarta, and South Kalimantan) using semi-structured interviews and open-ended questionnaires. Data were coded and analyzed thematically using NVivo 14 to identify emerging categories, patterns, and interconnections among teachers’ experiences and perceptions.

**Results:**

The findings indicate that social media effectively supports both collaborative and independent learning, enhances digital literacy, and increases motivation among students. Teachers reported that platforms such as WhatsApp and YouTube foster communication, creativity, and resource sharing. However, they also highlighted challenges related to unequal internet access, data privacy, and managing online behavior. Three key themes emerged: (1) social media as a catalyst for collaboration, (2) digital literacy development through authentic media interaction, and (3) the need for clear guidelines to maintain safety and equity.

**Conclusions:**

This study proposes the Social Media Networking (SMN) framework, offering practical steps for teachers to integrate social media into inclusive pedagogy. The SMN framework demonstrates that social media can function as a structured, participatory, and equitable learning strategy that strengthens inclusivity and engagement in Indonesian language classrooms.

## 1. Introduction

The rapid development of information and communication technology (ICT) has significantly transformed education in Indonesia. Recent surveys indicate that more than 80% of elementary school students actively use social media platforms such as WhatsApp, TikTok, and YouTube for daily communication and entertainment ( Fajar & Machmud, 2020). While these platforms create opportunities for more interactive and engaging learning, they also present critical challenges, particularly in inclusive elementary schools where students with special needs learn alongside their peers. Teachers often struggle to address distractions, exposure to inappropriate content, and unequal digital literacy among students. If these challenges remain unresolved, they risk widening the achievement gap and undermining the government’s mandate that all students, including those with special needs, must master Indonesian language skills.

This situation underscores a crucial gap, although social media is already embedded in children’s everyday lives, its systematic integration into Indonesian language learning strategies for inclusive classrooms has not been clearly articulated. By systematic integration, this study refers to the absence of a structured and evidence-based approach that guides teachers in (1) selecting appropriate platforms aligned with language learning goals, (2) designing inclusive activities that accommodate diverse learners’ abilities, (3) monitoring and supporting safe digital engagement, and (4) evaluating outcomes for continuous improvement. Currently, most uses of social media in classrooms remain driven by individual teacher initiative rather than by a consistent framework.

Prior studies support this observation. Barrot (2022) showed that teachers often adopt social media in language learning without aligning it to specific competencies, leading to inconsistent outcomes. Similarly, Jayanti (2023) found that while teachers recognize the potential of social media for enhancing communication skills, its application in inclusive classrooms remains unstructured and highly dependent on teachers’ individual creativity. These findings highlight the absence of a systematic model and reinforce the urgency of developing a framework that ensures social media functions as a supportive pedagogical tool rather than a source of distraction or inequality in achieving Indonesian language learning goals. This urgency is further reinforced by national education policies, such as the Minister of National Education Regulation No. 22 of 2006, which mandates the mastery of Indonesian communication skills for all students, and Regulation No. 70 of 2009, which emphasizes the right of students with special needs to access inclusive education. Without a clear and systematic strategy, these policy goals remain difficult to achieve in practice.

The use of social media among inclusive elementary school students raises several urgent concerns. Elementary-aged children are still in the early stages of cognitive and emotional development, limiting their ability to filter harmful online content. This makes them vulnerable to inappropriate exposure, cyberbullying, and internet addiction ( Hadi, 2024; Khairunisa et al., 2023). For students with special needs, these risks are even greater, often undermining their confidence and social participation. In addition, excessive engagement in online activities can reduce students’ focus on academic and social interactions in the classroom ( Andara et al., 2022; Fauziah et al., 2024; Humaira et al., 2021), which may further hinder learning progress in inclusive settings where individualized support is essential.

At the same time, prior studies show that social media platforms such as YouTube, TikTok, Instagram, and WhatsApp can support the development of speaking, listening, reading, and writing skills when used pedagogically ( Barrot, 2022; Prayogo & Sholikhati, 2021; Widiada et al., 2021). Social media has also been associated with improved communication skills ( Jayanti, 2023) and digital literacy ( Asafo-Adjei et al., 2023; Lambton-Howard et al., 2021; Neumann et al., 2022; Valencia, 2016; Valencia & Benavides, 2019; Xodabande, 2017; Zhuravleva et al., 2016). However, most of these studies focus on general classroom settings and have not specifically examined how social media can be systematically applied in inclusive Indonesian classrooms. Moreover, while some evidence suggests that guided use of social media may reduce the risk of addiction ( Gao et al., 2022), its integration into inclusive education remains unclear and requires systematic guidance.

Policy frameworks, such as the Regulation of the Minister of National Education (Permendiknas) No. 22 of 2006 on Indonesian language learning and Permendiknas No. 70 of 2009 on inclusive education, mandate that all students, regardless of their abilities, must acquire effective communication skills in Indonesian. Yet, teachers in inclusive classrooms continue to face difficulties in implementing strategies that accommodate diverse abilities and learning needs ( Rasmitadila et al., 2021, 2022, 2023). These challenges demonstrate both the significance of the problem and the urgency of developing a strategy that can guide teachers in making social media an effective pedagogical tool for inclusive Indonesian language learning.

In response to this gap, the purpose of this study is to develop a practical framework of social media based strategies for Indonesian language learning in inclusive elementary classrooms ( Humaira et al., 2019; Rasmitadila et al., 2023). More specifically, the study seeks to:

(1) Formulate a set of guidelines for teachers on integrating social media into daily Indonesian lessons;
(2) Identify the opportunities and challenges encountered by teachers and students in inclusive settings; and
(3) Evaluate how these strategies can enhance students’ Indonesian language competencies in listening, speaking, reading, and writing.

Therefore, this study is guided by the research question “How to systematically integrate social media into Indonesian language learning in inclusive elementary schools to overcome existing challenges and support the development of students’ language competencies?”

Beyond its practical contribution, this study also offers a theoretical perspective by extending the discussion of Social Media Networking (SMN) as a pedagogical model for inclusivity. By doing so, it enriches the literature on technology-enhanced learning in the context of inclusive classrooms and provides teachers with concrete, evidence-based steps for adapting social media to support the diverse strengths, weaknesses, and learning styles of all students.

## 2. Method

This study employed a qualitative case study approach to gain an in-depth understanding of how social media networking (SMN) can be integrated into Indonesian language learning in inclusive elementary schools ( Harrison et al., 2017; Sjoberg et al., 2020; Yin, 2016). The research was conducted in 15 inclusive elementary schools across five provinces in Indonesia (West Java, Central Java, East Java, Yogyakarta, and South Kalimantan). These provinces were selected because they represent different geographical, cultural, and educational contexts, thereby providing a broader perspective on inclusive education practices in Indonesia.

### 2.1 Units of study

The participants consisted of 15 classroom teachers (one from each school) selected through purposive sampling. The inclusion criteria were: (1) teachers currently teaching in inclusive classrooms with at least one student with special needs, and (2) teachers with direct experience in using or attempting to use social media in teaching. Of the 15 teachers, nine were female and six were male, with teaching experience ranging from 5 to 20 years (see Table 1). All participants had experience working with students with special needs in inclusive settings. This diversity in participant background contributed to the richness of perspectives across provinces.

**Table 1.** Participants’ demographics.

Province	Code respondent	Gender	Teaching experience (Years)
West Java	TWJ1	Female	8
	TWJ2	Female	6
	TWJ3	Male	15
Central Java	TCJ1	Female	12
	TCJ2	Female	14
	TCJ3	Female	20
East Java	TEJ1	Female	7
	TEJ2	Female	10
	TEJ3	Male	18
Yogyakarta	TY1	Female	15
	TY2	Female	8
	TY3	Male	20
South Kalimantan	TSK1	Female	5
	TSK2	Female	12
	TSK3	Female	7

### 2.2 Data collection methods

Data collection used a triangulation strategy, combining semi-structured interviews and questionnaires to enhance the credibility of findings.

1) **Semi-structured interviews** were conducted to collect qualitative data on teachers’ perceptions, experiences, challenges, and recommendations. The development of the interview guide followed a rigorous process. Initially, a list of 24 potential questions was compiled from a review of previous studies on social media in inclusive education and pedagogy ( Humaira et al., 2025). These questions were then grouped and refined into three main themes derived from the literature and the study’s conceptual framework: (1) perceptions of social media use in inclusive classrooms, (2) challenges and opportunities in integrating SMN into Indonesian language learning, and (3) recommendations for strategy development. To ensure validity and clarity, the draft interview guide was reviewed by three experts in inclusive education, Indonesian language, and elementary education. Based on their input, several questions were reformulated to be simpler and more culturally relevant, while repetitive items were removed. A pilot test with five teachers was also conducted, after which adjustments were made by adding follow-up questions on digital literacy and parental engagement, as these issues emerged significantly in the pilot test. The final interview guide was thus evidence-based and context-grounded, allowing for a rich yet focused exploration of the research questions ( Humaira et al., 2025). Interviews were conducted online (via Zoom) and/or in-person, depending on accessibility, and each lasted 45–60 minutes. With written consent, interviews were recorded using a digital audio recorder or Zoom’s recording feature and then transcribed verbatim for analysis.
2) The questionnaire, distributed via Google Forms, was designed to collect qualitative data from teachers ( Humaira et al., 2025). It consisted entirely of open-ended questions aimed at capturing teachers’ experiences, challenges, and expectations regarding the use of social media in Indonesian language learning within inclusive classrooms. Sample items included: *“How often do you use social media in teaching Indonesian?”* and *“What benefits or risks do you perceive for students with special needs?”*
  The initial pool of questions was adapted from prior studies on social media and language education ( Barrot, 2022; Jayanti, 2023), but these items were revised to suit the inclusive Indonesian context. Revisions considered specific cultural and educational factors, such as the prominence of WhatsApp and YouTube in Indonesia, the need for simple phrasing to accommodate varying levels of teachers’ digital literacy, and the inclusion of questions that explicitly addressed the learning experiences of students with special needs.
  A pilot test with five teachers was conducted to check clarity, feasibility, and contextual relevance. Although this number was small, these teachers were purposively selected from different provinces (Java and Kalimantan) to represent diverse school contexts. Their feedback was crucial: several questions were rephrased for clarity, one item was added to explore parental involvement in supervising students’ use of social media, and redundant or overlapping items were removed.
  Furthermore, the questionnaire underwent expert review by three specialists in inclusive education, Indonesian language, and elementary education, selected for their research expertise and practical experience in Indonesian classrooms. The experts recommended restructuring the sequence of items for better logical flow, refining the wording to reduce ambiguity, and ensuring that the prompts captured both opportunities and risks of social media use. These revisions strengthened the validity and contextual appropriateness of the instrument before its full administration.

The data collection process took place over four months, from August to November 2024. Questionnaires were distributed first (August–September) to gather baseline qualitative data, followed by interviews (September–November) to explore emerging themes in greater depth. Importantly, the analysis of questionnaire responses was conducted on a rolling basis rather than waiting until all responses were collected. This allowed the research team to identify patterns early and refine subsequent data collection. One such adjustment emerged from teachers’ open-ended responses, which consistently pointed to inequalities in digital literacy. For example, several teachers reported that students from lower-income families had limited access to devices or lacked the basic skills to use social media features such as uploading files or creating videos. Recognizing the importance of this issue, the research team modified the interview guide by expanding certain probes. Specifically, additional follow-up questions were included to explore (1) how teachers supported students with low digital literacy, (2) what strategies were used in classrooms with high variation in digital skills, and (3) whether peer mentoring or parental involvement played a role. These iterative adjustments illustrate the study’s flexible design and ensured that the interviews addressed not only the initially planned themes but also new, context-specific issues that emerged from the questionnaire data.

### 2.3 Data processing

All interview recordings were transcribed verbatim, and open-ended questionnaire responses were entered into NVivo 14 for analysis. Data were anonymized by assigning codes based on province names (e.g., TWJ1, TCJ1, TEJ1, TY1, TSK1) to replace participants’ names and schools. Transcripts and datasets were stored in a restricted-access Google Drive folder, which was available only to the research team. To further ensure confidentiality and compliance with ethical standards, the folder was secured with password protection and data encryption **.** Data integrity was verified by cross-checking transcripts with recordings and validating questionnaire entries. These measures minimized the risk of unauthorized access and safeguarded the privacy of all participants throughout the research process.

### 2.4 Data analysis

Thematic analysis ( Clarke & Braun, 2013) was used to identify recurring patterns. This process involved data familiarization, initial coding, theme construction, theme review, and final reporting. Coding was conducted independently by two researchers; discrepancies were discussed until consensus was reached.

Questionnaire data were analyzed through open-ended responses (e.g., descriptions of teachers’ experiences, expectations, and perceived challenges) and coded thematically using NVivo 14. The coding process followed Clarke & Braun’s (2013) six steps of thematic analysis (familiarization, initial coding, theme discovery, theme review, defining/naming themes, and reporting). These open-ended responses provided nuanced insights into teachers’ practices and attitudes. NVivo 14 was used to manage qualitative data and visualize coding structures.

### 2.5 Techniques to increase trustworthiness

Several strategies were implemented to strengthen trustworthiness and credibility. First, triangulation of interviews and questionnaires allowed for cross-validation of findings. Second, member checking was conducted by sharing initial results with three participants to ensure the accuracy and resonance of interpretations. While the number may seem limited, this was due to practical constraints such as teachers’ limited availability during the academic semester and the need to avoid imposing additional workload. The three teachers were purposively selected to represent different provinces and teaching experiences, ensuring that their feedback reflected diverse perspectives. Their input confirmed the clarity of themes and provided suggestions for refining the interpretation of digital literacy challenges. Third, an audit trail documented coding decisions and analytical memos, ensuring transparency. Fourth, double coding by two researchers reduced bias and increased reliability. Fifth, anonymization and confidentiality measures maintained ethical standards.

### 2.6 Ethical considerations

This study complied with the principles of the Declaration of Helsinki and was approved by the Research Ethics Committee of Universitas Djuanda (Approval No. 002/LPPM/KEP/VIII/2024). All participating teachers provided written informed consent (No. 002/IC/LPPM/XI/2024) after receiving information on the study’s objectives, procedures, and their right to withdraw at any time. To ensure confidentiality, all personal identifiers were removed and replaced with province-based codes (e.g., TWJ1 for West Java, TCJ1 for Central Java). Data were securely stored in a password-protected, encrypted Google Drive folder accessible only to the research team. These measures ensured participant privacy, data integrity, and adherence to the highest ethical standards in educational research.

## 3. Results and discussion

This study involved 15 classroom teachers from inclusive elementary schools across five provinces ( Table 1): West Java (3), Central Java (3), East Java (3), Yogyakarta (3), and South Kalimantan (3). Among these teachers, nine were female and six were male, with teaching experience ranging from 5 to 20 years. All of them had experience teaching students with special needs in inclusive classrooms. The diversity of these teachers’ backgrounds provided rich insights and a broad perspective on how social media is utilized across different regional and school contexts.

Based on the analysis of interview and questionnaire data, several major themes emerged that highlight both the potential and the challenges of using social media in inclusive Indonesian language learning. These themes are: (1) the effectiveness of social media in supporting collaborative learning, (2) the role of social media in enhancing digital literacy skills, and (3) the challenges of maintaining student safety and privacy on online platforms ( Figure 1).

**Figure 1.**
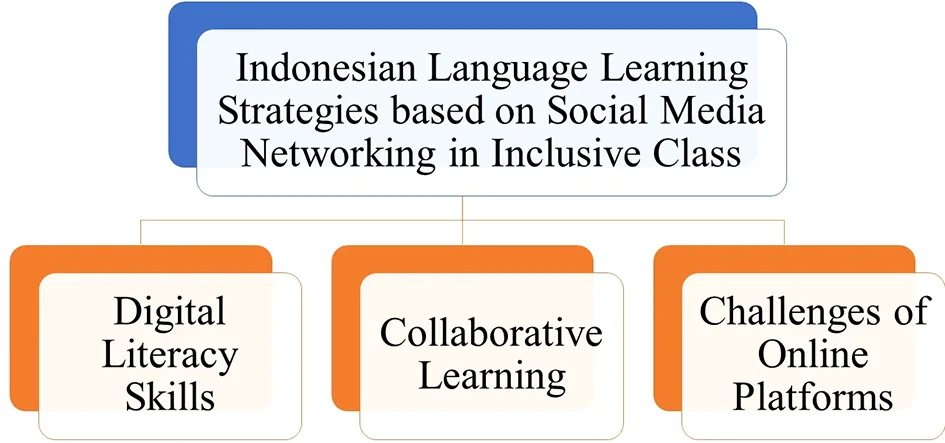
Indonesian language learning strategies based on social media networking in inclusive classes.

### 3.1 Effectiveness of social media in supporting collaborative learning (Theme 1)

Social media has proven effective in supporting collaborative learning in inclusive elementary school environments ( Bozanta & Mardikyan, 2017; Damayanti & Nuzuli, 2023; Hajarudin, 2023; McPherson, 2018). Teachers and students reported that platforms such as WhatsApp, Google Classroom, TikTok, and YouTube enabled them to interact, share ideas, and work together on Indonesian language learning projects ( Figure 2). Students felt more engaged because they could collaborate in real time both inside and outside the classroom ( Boruzie et al., 2024; Klang et al., 2020).

**Figure 2.**
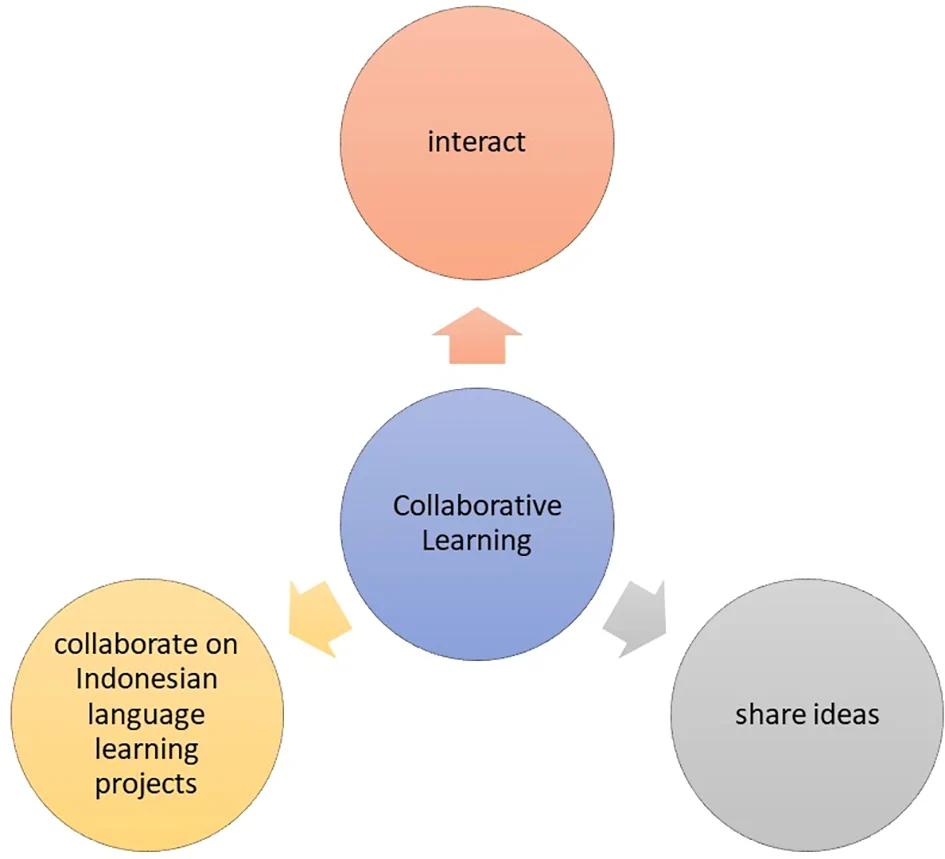
Theme: Collaborative learning.

Teachers highlighted that social media facilitated smoother communication between students with different needs. As one teacher explained:

*“Through WhatsApp groups, students who are usually quiet in class became more active in asking questions and sharing assignments.”*

(TCJ1, 12 years teaching experience)

Another teacher noted how social media helped break down barriers for students with special needs:

*“Students with special needs could send voice notes instead of writing long texts, so their friends understood their ideas better.”*

(TWJ1, 8 years teaching experience)

In this way, “smoother communication” referred to providing alternative ways for participation, enabling collaboration despite differences in ability.

Social media also supported asynchronous collaboration, allowing students to engage with materials beyond regular class hours. One teacher described:

*“Some students reviewed the lesson videos at night and then discussed them the next morning. They didn’t have to understand everything at once in class.”*

(TY1, 15 years teaching experience)

This flexibility reduced pressure for students who faced barriers such as shyness, social anxiety, or physical limitations, enabling them to contribute at their own pace.

Social media provides significant flexibility because it allows students to interact and collaborate without being limited by classroom boundaries or time. In inclusive schools, this flexibility is especially important to accommodate students with different backgrounds and abilities, including those with special needs. By using social media, students can access learning materials, interact with teachers, and discuss with classmates at any time. This helps students learn at their own rhythm, especially those who require more time to understand the material ( Demetriou, 2023; Khanna & Bhola, 2023).

Social media also gives students opportunities to review materials, complete assignments within deadlines, or ask teachers questions outside school hours. Teachers, in turn, can apply differentiated approaches by providing content in various formats (video, text, images, or audio) to suit students’ diverse learning needs ( Demetriou, 2023). Collaboration was further observed when students gave feedback to one another and discussed lesson materials, which was particularly helpful for those struggling to understand the content.

Despite these benefits, challenges remained, such as the lack of active participation among students unfamiliar with technology or those with limited access. Teachers therefore play a crucial role in monitoring, facilitating, and offering additional support to ensure all students, including those with special needs, can participate optimally. With a proactive approach, these challenges can be addressed, allowing social media–based collaborative learning to become more inclusive and effective.

### 3.2 The role of social media in improving digital literacy skills (Theme 2)

The use of social media in Indonesian language learning in inclusive elementary schools has strong potential to enhance students’ digital literacy skills ( Erazo-Moreno et al., 2024; Riga et al., 2021) ( Figure 3). Through social media, students not only socialize but also practice searching for information, reading and writing critically, and understanding various digital text formats. These activities support their Indonesian language learning while simultaneously fostering essential technology skills needed for the future.

**Figure 3.**
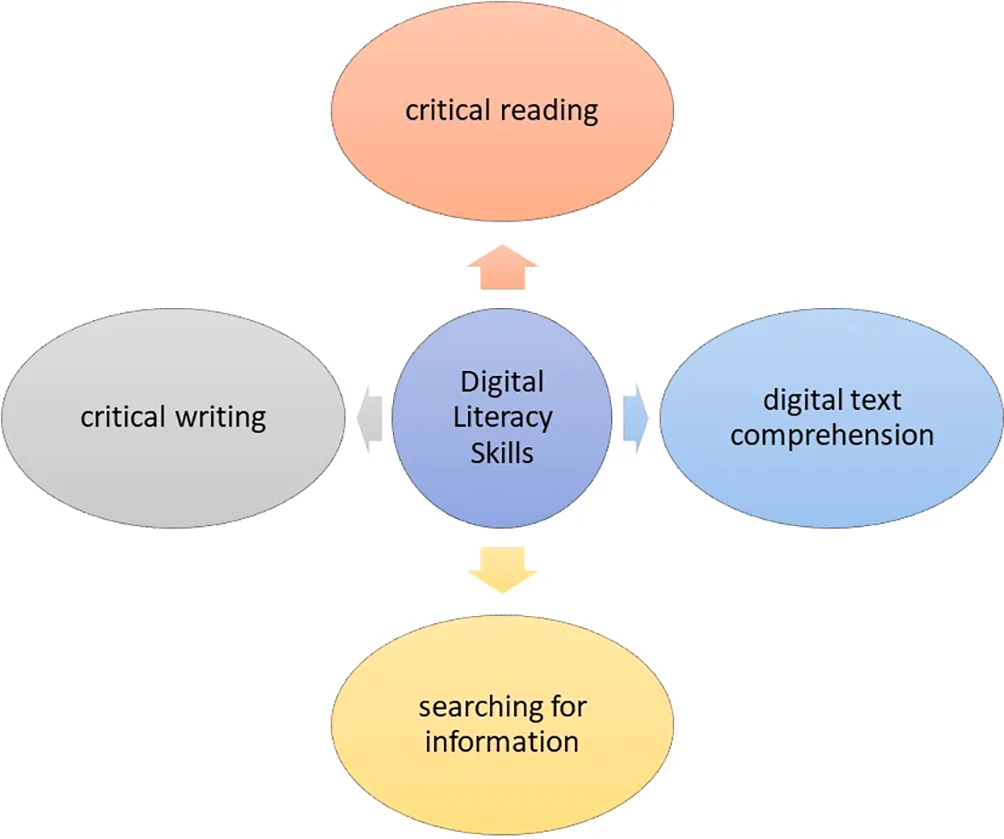
Theme: Digital literacy skills.

Social media provides students with access to a wide and diverse range of information. Tasks such as creating video content, writing short articles, or engaging in online discussions train students to manage information, evaluate the validity of sources, and communicate effectively in digital contexts ( Baxter & Reeves, 2023; Gillett‐Swan & Sargeant, 2018). In this way, social media–based learning enriches Indonesian language skills through interactive activities like video production, writing captions, commenting on peers’ work, or participating in digital discussions. At the same time, students build technological competence by productively using digital devices and online platforms ( Bastian et al., 2023; Riga et al., 2021).

Teachers observed that these tasks such as producing short TikTok videos or writing captions helped students develop digital literacy. However, it is important to note that these skills were reported by teachers based on classroom observations, not directly measured by researchers. For example:

*“When I asked students to make a TikTok video explaining a short story, they learned not only to speak clearly but also to edit the video and choose appropriate visuals.”*

(TEJ2, 10 years teaching experience)

Despite these benefits, differences in digital literacy were evident, particularly among students from economically disadvantaged families:

*“Some children didn’t have stable internet access, so they only borrowed their parents’ phones occasionally. They were left behind in group tasks.”*

(TSK3, 7 years teaching experience)

Such students struggled not only with access but also with basic digital skills, such as uploading files or using simple editing tools. These disparities highlight the inequality of opportunities in inclusive classrooms and the need for additional teacher support to ensure equitable participation.

### 3.3 Challenges on online platforms (Theme 3)

Although social media offers many learning benefits, one of the main findings of this study is the concern regarding student safety ( Figure 4). Teachers and parents emphasized that elementary school students are vulnerable to risks such as cyberbullying and exposure to inappropriate content ( Ananda & Marno, 2023; Hafiar et al., 2020; Wong et al., 2022).

**Figure 4.**
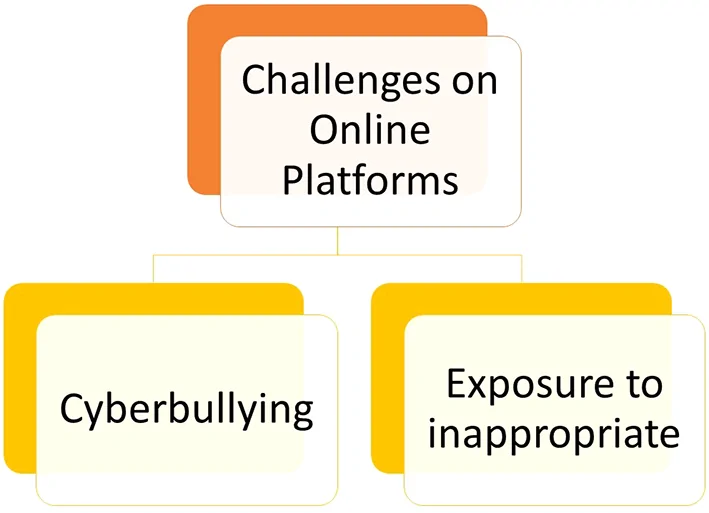
Theme: Challenges of online platforms.

While social media enhances interaction and collaboration, it also brings significant risks. These risks are particularly relevant in Indonesia, where elementary school students are still developing emotionally and socially, making them more susceptible to negative influences from the online environment. Cyberbullying defined as intimidation or hurtful behavior through digital media such as social networks, text messages, or other online platforms was a major concern ( Evangelio et al., 2022). Teachers shared that incident of cyberbullying had already occurred:

*“One student uploaded a classmate’s photo without permission, and it became a joke in the group. The child felt very embarrassed.”*

(TWJ2, 6 years teaching experience)

In addition to cyberbullying, teachers and parents raised concerns about exposure to inappropriate content, such as violent or irrelevant videos that appeared on students’ TikTok feeds. Despite existing user age restrictions and content regulations, many children were still able to access harmful materials, including violence, pornography, or misleading information ( Hafiar et al., 2020). These experiences reinforced the need for stronger supervision in inclusive classrooms.

Collaboration between teachers and parents was described as crucial but often informal and reactive:

*“When we found a problem, I immediately called the parents to discuss how to handle it together.”*

(TCJ2, 14 years teaching experience)

### 3.4 Proposed framework: Social Media Networking (SMN) Strategy for inclusive Indonesian language learning

To directly address the challenges identified in this study and to fulfill its stated objective, a Social Media Networking (SMN) framework is proposed for application in inclusive Indonesian language classrooms. The framework is structured into four interconnected stages: planning, implementation, monitoring and support, and evaluation and reflection.

**3.4.1 Planning**

In this stage, teachers align Indonesian language learning objectives (listening, speaking, reading, and writing) with the choice of social media platforms that are both familiar and accessible to students. The findings from both interviews and questionnaires confirmed that teachers frequently selected WhatsApp for text and voice communication, TikTok and YouTube for video-based activities, and Google Classroom for assignment management. Similar findings are reported by Barrot (2022), who emphasized that social media platforms are effective in supporting language acquisition when teachers carefully match learning goals with platform features. This alignment ensures that technology becomes an enabler rather than a distraction, particularly for students with special needs who require multiple modes of content delivery.

**3.4.2 Implementation**

Learning activities should be designed to allow both synchronous (real-time) and asynchronous (flexible-time) participation. Teachers in this study reported that asynchronous activities such as reviewing videos at home or sending voice notes gave students with shyness, anxiety, or physical limitations more time and space to contribute meaningfully. As one teacher explained:

*“Some students who rarely spoke in class became more confident when asked to record a short video or send a voice message through WhatsApp.”*

(TWJ3, 15 years teaching experience)

This resonates with Demetriou (2023) and Khanna & Bhola (2023), who found that flexible participation is particularly beneficial in inclusive classrooms, as it respects individual learning rhythms. The combination of synchronous discussions and asynchronous submissions thus allows differentiated participation, ensuring that all students can demonstrate their abilities in ways that suit them best.

**3.4.3 Monitoring and support**

The triangulation of data from interviews and questionnaires revealed strong concerns about cyberbullying and exposure to inappropriate content. Teachers consistently emphasized the need for closer supervision. For example:

*“When students used TikTok, I noticed that some unrelated videos appeared, so I had to guide them on how to filter what was useful.”*

(TCJ3, 20 years teaching experience)

In this framework, teachers and parents must collaborate actively to monitor students’ online activities, both to ensure safety and to promote responsible digital behavior. This finding is consistent with Wong et al. (2022), who stressed the moderating role of inclusive norms in minimizing online risks. Furthermore, the data showed that students from economically disadvantaged families often lacked adequate digital skills, requiring additional scaffolding such as tutorials or peer mentoring. Teachers’ role in providing this support is critical to reducing inequality in participation.

**3.4.4 Evaluation and reflection**

The final stage focuses on evaluating students’ engagement and achievements in Indonesian language competencies (listening, speaking, reading, and writing). In this study, teachers reported improvements in oral participation, writing fluency, and confidence when students were encouraged to use social media creatively (e.g., creating video summaries of texts). However, teachers also admitted that these observations were mostly qualitative and based on classroom monitoring, suggesting the need for further empirical measurement in future research. The evaluation stage also involves gathering feedback from students and parents, which provides a reflective loop to improve the integration of SMN in subsequent learning cycles. This aligns with Clarke & Braun (2013), who emphasize reflexivity as a key element of thematic analysis in educational contexts.

From a practical perspective, the SMN framework provides teachers with clear, actionable steps that directly address the opportunities and challenges observed in inclusive classrooms. By embedding structured planning, flexible implementation, collaborative monitoring, and continuous evaluation, the framework offers a pathway for teachers to leverage social media as a meaningful pedagogical tool rather than a source of distraction. From a theoretical perspective, this study contributes by conceptualizing SMN not merely as a digital platform, but as a pedagogical model for inclusivity. It highlights the dual function of social media as both a space for language learning and a medium that fosters equity through differentiated participation. This addresses the research gap identified in the introduction, as previous studies have examined social media for language learning in general ( Barrot, 2022; Valencia & Benavides, 2019), but few have explored its systematic application in inclusive Indonesian classrooms.

Finally, the triangulation of interviews and questionnaires reinforces the validity of this framework. Teachers’ narratives provided rich, contextualized evidence, while questionnaire data confirmed patterns across multiple schools and provinces. This consistency across sources demonstrates that the proposed SMN strategy is both evidence-based and contextually grounded, making it a valuable contribution to the field of inclusive education.

## 4. Conclusion

This study set out to design strategies for Indonesian language learning using Social Media Networking (SMN) in inclusive elementary schools. The findings indicate that SMN can enhance collaboration, digital literacy, and accessibility, especially for students with special needs, thereby strengthening competencies in listening, speaking, reading, and writing. At the same time, challenges such as cyberbullying, exposure to inappropriate content, and unequal participation highlight the importance of careful guidance and monitoring. The main contribution of this study lies in the proposed SMN framework, which provides teachers with structured, practical steps for integrating social media into inclusive Indonesian language classrooms. This framework is unique in that it combines both pedagogical strategies and inclusivity principles, ensuring that diverse learners can participate according to their strengths and limitations. The conclusions of this research are primarily applicable to inclusive elementary schools in Indonesia, reflecting the specific context of national policies, cultural practices, and digital access. However, the strategies and framework developed here also have the potential to be adapted in other educational settings where social media is widely used and where inclusivity is a key concern. By making these adaptations, the findings can inform broader discussions on how technology can support equitable learning worldwide.

## Ethics and consent

The research and community service department of Universitas Djuanda has approved this research with the certificate number: 002/LPPM/KEP/VIII/2024 on August 4th 2024. The researcher gave a letter of approval has also been given by the researcher to all respondents. Written consent to participate from the respondent was obtained in accordance with document No. 002/IC/LPPM/XI/2024 on November 28th 2024. Respondents gave their consent without force from anyone. Subse quently, in order to protect the rights and privacy of the respondents, all forms of data acquired will remain confidential

## Data Availability

Figshare: Thematic Analysis: Social Media Networking (SMN) Model for Inclusive Indonesian Language Learning. This project contains the following underlying data
• Thematic Analysis: Social Media Networking (SMN) Model for Inclusive Indonesian Language Learning Doi: https://doi.org/10.6084/m9.figshare.30423688.v1
• Participants’ Demographics: Social Media Networking (SMN) Model for Inclusive Indonesian Language Learning Doi: https://doi.org/10.6084/m9.figshare.30423940.v1 Data are available under the terms of the Creative Commons Attribution 4.0 International license (CC-BY 4.0). Figshare: Teachers’ Perspectives on Social Media Networking for Indonesian Language Learning in Inclusive Elementary Schools: A Multi-Method Case Study This project contains the following underlying data
• Teacher Interview Questions (Social Media Networking) Doi: https://doi.org/10.6084/m9.figshare.30361285.v3 ( Humaira et al., 2025) • Teacher Questionnaire: Social Media Networking (SMN) Model for Inclusive Indonesian Language Learning Doi: https://doi.org/10.6084/m9.figshare.30423211.v1 ( Humaira et al., 2025) • Statement Letter Ethical Consideration (Social Media Networking) Doi: https://doi.org/10.6084/m9.figshare.30423250.v1 Data are available under the terms of the Creative Commons Attribution 4.0 International license (CC-BY 4.0).
