# Supplementary material for: Teachers’ Perspectives on Social Media Networking for Indonesian Language Learning in Inclusive Elementary Schools: A Multi-Method Case Study
Source: F1000Res. 2026 Jul 10;14:1304. Originally published 2025 Nov 24. [Version 2] doi: 10.12688/f1000research.172079.2 (PMC13648950; doi:10.12688/f1000research.172079.2)
Supplement: Peer review history

# Reviewer report: Reviewer response for version 2

**DOI:** 10.5256/f1000research.195293.r516345

**Published:** 2026-09-02

**Authors:** Mahmud Fasya

**Affiliations:** 1 Universitas Pendidikan Indonesia, Bandung, West Java, Indonesia

**Overall Review**

 The manuscript, entitled **“Teachers’ Perspectives on Social Media Networking for Indonesian Language Learning in Inclusive Elementary Schools: A Multi-Method Case Study,”** addresses a timely and potentially significant topic at the intersection of social media, Indonesian language education, and inclusive elementary education. The focus on inclusive schools is particularly relevant because the pedagogical use of digital media may simultaneously create opportunities for differentiated participation and introduce new forms of digital inequality and online risk.

 The manuscript is generally well organized, and the authors have made considerable efforts to provide information regarding participant selection, instrument development, data collection, thematic analysis, trustworthiness, and ethical procedures. The inclusion of teachers from 15 inclusive elementary schools across five Indonesian provinces also provides potentially valuable contextual variation. However, several substantial conceptual, methodological, analytical, and interpretive issues need to be addressed. In its current form, the manuscript tends to make claims that extend beyond what can be adequately supported by the qualitative evidence.

 I therefore recommend **Major Revision**.

**Major Comments**

**Conceptual clarity of “Social Media Networking” (SMN)**

 The most fundamental conceptual issue concerns the term **“Social Media Networking (SMN)”**. The manuscript does not sufficiently distinguish “social media networking” from more commonly used concepts such as *social media use*, *social media integration*, or *social media-based learning*. Define SMN precisely and explain what constitutes SMN in this study. The term “networking” may imply the formation and maintenance of social networks or relationships. In contrast, much of the manuscript appears to concern the pedagogical use of platforms such as WhatsApp, TikTok, YouTube, and Google Classroom.

 Define SMN precisely and explain what constitutes SMN in this study. If the research primarily examines teachers’ use or integration of social media for Indonesian language learning, a more precise terminology may be appropriate. This conceptual clarification is important because the terminology is used consistently throughout the title, methodology, findings, framework, and conclusion, and therefore affects the construct validity of the entire manuscript.

**Alignment between the title, research design, and empirical evidence**

 The title identifies the study as a **“Multi-Method Case Study”** and emphasizes **“Teachers’ Perspectives.”** However, the methodology and findings do not yet provide sufficient evidence to clarify what constitutes the “case” or how the multi-method design is structured. Revise the methods section to define the study design more clearly and align it with the title. Clarify whether the study is a single case, multiple cases, or a multisite qualitative study, and explain how the design fits the title.

 The study involved 15 teachers from 15 schools across five provinces. Clarify whether this constitutes a single case, multiple cases, or a multisite qualitative study. If each school is treated as a case, describe the design explicitly as a multiple-case study. Explain how this participant structure supports the study design.

 Likewise, the manuscript should explain more clearly how the different data sources were integrated within the study design. At present, the empirical findings are dominated by interview quotations, even though the authors claim that both interviews and questionnaires were used and triangulated. The methods section should clarify how these sources were combined. Clarify how each source contributes to the overall design and how the integration was carried out.

**Inconsistency in the description of data collection**

 There is a significant inconsistency between the opening methodological description and the detailed data collection section. The opening section states that the multi-methods design involved **semi-structured interviews, classroom observations, and document analysis**. In contrast, the subsequent methodology describes **semi-structured interviews and open-ended questionnaires** as the actual data sources. Align these descriptions throughout the methods section to present one clear design.

 Clarify whether classroom observations and document analyses were actually conducted. If they were, report their procedures, sources, duration, sampling, and analytical treatment. If they were not conducted, correct the opening description so it matches the methods section.

 This is more than a stylistic issue because it directly affects the credibility and reproducibility of the research design. Present a consistent account of all data collection procedures so the methods section is clear.

**Justification and role of the qualitative questionnaire**

 The manuscript describes the questionnaire as a qualitative data-collection instrument and indicates that it consisted entirely of open-ended questions. This approach is possible, but its role within the overall design requires further explanation, especially in relation to the interviews. Clarify why the questionnaire was included in the study design and how it functioned alongside the interviews. Explain how it complements the interviews within the design.

 Report the number of questionnaire responses, explain whether all 15 teachers completed the questionnaire, clarify the relationship between questionnaire responses and interviews, and explain why an open-ended questionnaire was preferred as a complementary method. Also explain how the questionnaire data were used in the analysis and how this supported the study design. Describe the role of each source clearly.

 Furthermore, an item such as “How often do you use social media in teaching Indonesian?” potentially generates frequency information that may be descriptive or quasi-quantitative. The authors should clarify how such responses were treated analytically and whether they were used to support the interview findings, provide additional context, or both. This clarification belongs in the methods section. State how this item fits the study design.

**Sampling adequacy and participant selection**

 The study includes 15 teachers, one from each school, selected purposively. A sample of 15 participants can be defensible for qualitative research, but the manuscript needs a stronger rationale for sample adequacy within the study design. Explain how the sample size fits the design and participant structure.

 Explain how the schools and teachers were selected, why one teacher per school was considered sufficient, and whether sample adequacy was determined through saturation, information power, or another qualitative rationale. Add background details that clarify the participant selection within the study design. Describe how selection decisions shaped the case structure.

 The rationale for selecting five provinces should likewise be presented more carefully. The claim that these provinces provide a “broader perspective” should not imply national representativeness unless the sampling strategy supports such a conclusion. Clarify how this geographic spread supports the study design. Explain what role the provinces play in defining the cases.

**Relationship between pilot testing and the final sample**

 The manuscript states that five teachers participated in pilot testing of the interview guide and questionnaire. Explicitly state whether these five teachers were included in or excluded from the final sample to clarify the study design. Explain how their participation affects the final sample structure.

 If they were included, address the potential influence of prior exposure to the instrument. If they were excluded, state this explicitly to keep the study design clear. Clarify the final sample composition and its relation to the pilot phase.

**Data analysis requires greater methodological transparency**

 The authors employ thematic analysis and state that two researchers independently coded the data before resolving discrepancies through discussion and consensus. This is appropriate, but the analytical process within the study design remains insufficiently detailed. Clarify the analytic design more fully. Explain how the analytic procedures fit the multi-method design.

 Clarify whether the thematic analysis was inductive, deductive, or hybrid; how the initial codes were generated; whether interview and questionnaire datasets were initially analyzed separately; how themes were developed; and how the two datasets were subsequently integrated. State these points clearly in the methods section to make the study design transparent. Explain the sequence of analysis within the design.

 The use of NVivo is useful for data management, but software use itself does not establish analytical rigor. Give greater attention to the interpretive process rather than simply reporting the software employed. Emphasize analysis procedures over software to clarify the study design. State how the software supported the analytic workflow.

**Triangulation needs to be demonstrated rather than asserted**

 The manuscript repeatedly states that interviews and questionnaires were triangulated to enhance credibility. However, the findings do not clearly demonstrate how the two sources converged, complemented each other, or produced divergent evidence within the study design. Show this more explicitly. Explain how triangulation was built into the design.

 Show more explicitly how triangulation contributed to the development of the themes. A matrix or other transparent representation of the relationship between the two data sources could be useful for clarifying the study design. Present the integration process so the design is easier to follow.

**The findings need stronger empirical grounding**

 The three themes—collaborative learning, digital literacy, and safety/privacy—are clearly presented. However, several claims appear broader than the evidence presented.

 For example, statements such as **“social media has proven effective”** imply demonstrated effectiveness, whereas the study primarily relies on teachers’ perceptions, reflections, and qualitative observations. The manuscript itself acknowledges that improvements in students’ language skills were not established through standardized assessments or direct measures of student achievement.

 The authors should therefore consistently distinguish between **perceived benefits** and **empirically demonstrated effects**.

 Terms such as “teachers reported,” “participants perceived,” and “the findings suggest” would be more appropriate than causal formulations such as “SMN improves” or “SMN enhances,” unless supported by direct evidence.

**Greater attention to divergent teacher perspectives is needed**

 Because the article is explicitly framed around **teachers’ perspectives**, the analysis should provide greater evidence of variation within the participant group.

 The current presentation relies heavily on selected quotations but does not indicate how widespread particular views were among the 15 participants. The authors should consider whether there were contrasting perspectives, negative experiences, differences between provinces, differences in teaching experience, or differences related to teachers’ digital competence.

 The analysis would be considerably stronger if it demonstrated not only what teachers agreed upon but also where their experiences and interpretations diverged.

**The dual role of social media deserves deeper analysis**

 One of the most potentially important findings is the tension between social media as a mechanism of inclusion and social media as a source of exclusion.

 The findings suggest that social media can provide alternative forms of participation—for example, voice notes and asynchronous activities—while students with limited device access or digital literacy may be disadvantaged.

 This tension could constitute a significant contribution of the article. Rather than presenting digital inequality merely as a challenge, the authors could theorize more explicitly how the same technology may simultaneously facilitate inclusion for some learners and reproduce exclusion for others.

 This would make the discussion more sophisticated and potentially more valuable to an international readership.

**The discussion needs to move beyond confirmation of previous studies**

 The discussion frequently follows a pattern in which a finding is presented and then linked to previous literature. While such contextualization is useful, the discussion should go further by asking how the findings **confirm, extend, complicate, or challenge** existing scholarship.

 In particular, the authors should explain what is specifically revealed by the Indonesian inclusive elementary-school context. The discussion should avoid becoming a sequence of statements that the findings are “consistent with” previous studies.

**The proposed SMN framework requires methodological justification**

 The transition from the three empirical themes to the proposed **four-stage SMN framework**—planning, implementation, monitoring and support, and evaluation and reflection—is potentially useful. Still, the manuscript does not adequately explain how the framework was developed.

 The authors should clarify:

- whether the framework was derived inductively from the thematic findings;
- whether it was based on an existing theoretical framework;
- how the four stages were constructed;
- whether teachers or experts reviewed the framework;
- whether it was piloted or implemented in classrooms; and
- what evidence supports its effectiveness.

Without such information, the framework appears more like a set of practice-oriented recommendations than a validated pedagogical model.

**The claims concerning the framework’s validity are too strong**

 The statement that triangulation demonstrates that the proposed strategy is “evidence-based” and “contextually grounded” is potentially acceptable in a limited sense. Still, the manuscript goes further by implying that triangulation establishes the validity of the strategy.

 Triangulation can strengthen the credibility of qualitative interpretations, but it does not by itself validate the effectiveness of a newly proposed pedagogical strategy.

 The authors should therefore distinguish between:

**credibility of the qualitative findings → basis for proposing the framework → validation/effectiveness of the framework.**

 These are different levels of inference.

**Theoretical contribution is currently overstated**

 The manuscript claims that the study contributes by conceptualizing SMN as a pedagogical model for inclusivity. This may be an interesting proposition, but the current evidence does not yet demonstrate a sufficiently developed theoretical contribution.

 The authors should distinguish clearly between a **practical framework**, a **conceptual proposition**, and a **theoretical contribution**. If the framework is primarily intended to guide teachers’ practice, the contribution should be presented accordingly.

**Conclusions should be more closely aligned with the evidence**

 The conclusion contains several claims that are stronger than the study design permits. In particular, the statement that SMN “can enhance” collaboration, digital literacy, and accessibility and that it strengthens listening, speaking, reading, and writing competencies suggests objectively demonstrated effects. The qualitative evidence does not fully support these claims.

 The conclusion should instead emphasize teachers’ reported perceptions and experiences and acknowledge that direct measures of student learning were not used.

 Similarly, the claim that the framework is “unique” should either be supported through systematic comparison with existing frameworks or removed.

 The manuscript appropriately limits the applicability of the findings to inclusive elementary schools in Indonesia, but the subsequent claim that the framework may inform discussions “worldwide” should be moderated. Transferability to other settings should be presented as a possibility requiring contextual adaptation and further empirical investigation.

**Minor Comments**

1. The terminology surrounding *social media*, *social networking*, and *Social Media Networking (SMN)* should be standardized throughout the manuscript.
2. The authors should ensure that all platforms identified as social media are conceptually justified. In particular, the status of Google Classroom within the SMN construct should be clarified.
3. The manuscript would benefit from more explicit definitions of “inclusion,” “digital literacy,” “accessibility,” and “student engagement.”
4. The distinction between teachers’ observations and researchers’ observations should be made explicit. This is particularly important because the manuscript sometimes uses “classroom observations” in a way that may suggest systematic researcher observation, whereas the methodology does not adequately document such a procedure.
5. Claims concerning cyberbullying and inappropriate content should indicate whether they represent isolated incidents, recurring experiences, or patterns reported by multiple participants. The current evidence includes illustrative quotations but does not sufficiently establish prevalence.
6. The ethical section is relatively strong, particularly regarding ethics approval, informed consent, confidentiality, and data security. However, the relevance and rationale for invoking the Declaration of Helsinki in this educational research context could be explained more carefully.

**Overall Recommendation**

 The manuscript addresses an important and contemporary issue and has the potential to make a useful contribution to research on inclusive education and digital language pedagogy. Its strongest potential contribution lies not simply in demonstrating that social media can support learning, but in revealing the **dual and potentially contradictory role of social media: it may facilitate differentiated participation and collaboration while simultaneously creating digital inequality, privacy concerns, and safety risks**.

 However, the manuscript currently requires substantial revision in order to establish stronger alignment among its conceptual framework, research design, empirical findings, proposed strategy, and conclusions. In particular, the authors should resolve the inconsistency concerning the data sources, clarify the case-study design, strengthen the explanation of multi-methods integration and thematic analysis, provide greater evidence for the diversity of teachers’ perspectives, and moderate claims concerning effectiveness and framework validity.

 Most importantly, the proposed SMN framework should either be supported by a transparent development and validation process or be repositioned as a **practice-oriented framework derived from the qualitative findings**, rather than presented as a validated pedagogical model.

**Recommendation: Major Revision.**

Is the work clearly and accurately presented and does it cite the current literature?

Partly

If applicable, is the statistical analysis and its interpretation appropriate?

Not applicable

Are all the source data underlying the results available to ensure full reproducibility?

No

Is the study design appropriate and is the work technically sound?

Partly

Are the conclusions drawn adequately supported by the results?

Partly

Are sufficient details of methods and analysis provided to allow replication by others?

Partly

Reviewer Expertise:

I am a doctor of philosophy. My research focuses on language, culture & society, particularly on the exploration of Sundanese local knowledge recorded in language use.

I confirm that I have read this submission and believe that I have an appropriate level of expertise to confirm that it is of an acceptable scientific standard, however I have significant reservations, as outlined above.

# Reviewer report: Reviewer response for version 1

**DOI:** 10.5256/f1000research.189766.r446072

**Published:** 2026-01-29

**Authors:** Minsih Minsih

**Affiliations:** 1 Universitas Muhammadiyah Surakarta, Surakarta, Indonesia

This article explores the use of Social Media Networking (SMN) for Indonesian language learning in inclusive elementary schools. The study is highly relevant to contemporary educational challenges and presents a clear, contextually grounded framework that integrates inclusivity, digital literacy, and language learning.

 The manuscript demonstrates several strengths, including a solid policy and theoretical foundation, a transparent and rigorous qualitative methodology, and rich empirical data supported by teachers’ narratives. The proposed SMN framework—comprising planning, implementation, monitoring, and evaluation—is practical, well structured, and offers clear implications for teachers working in inclusive educational settings. The integration of national education policies with recent international literature further enhances the study’s scholarly and practical significance.

 Nevertheless, some aspects could be strengthened. First, the use of the term multi-method requires clarification, as the study relies exclusively on qualitative data; describing it as a multi-source qualitative case study would improve methodological precision. Second, although the SMN framework is practically valuable, its theoretical novelty could be articulated more explicitly by positioning it in relation to existing technology-enhanced learning models. Third, the reported improvements in students’ language skills are based on teachers’ perceptions; this limitation should be emphasized, alongside recommendations for future empirical validation or mixed-methods research.

 Overall, this is a well-written and meaningful contribution to the literature on inclusive education and technology-enhanced language learning. With minor revisions to clarify the methodology and strengthen the articulation of its theoretical contribution, the article is worthy of indexing.

 References:

 Valencia JAÁ: Language views on social networking sites for language learning: the case of Busuu. Comput. Assist. Lang. Learn. 2016; 29(5): 853–867. Publisher Full Text Valencia JAÁ, Benavides AF: Using social networking sites for language learning to develop intercultural competence in language education programs. J. Int. Intercult. Commun. 2019; 12(1): 23–42. Publisher Full Text Ananda ER, Marno M: Analisis dampak penggunaan teknologi media sosial terhadap perilaku bullying di kalangan siswa sekolah dasar ditinjau dari nilai karakter self-confident siswa dalam konteks pendidikan. EDUKATIF: Jurnal Ilmu Pendidikan. 2023; 5(5). Andara S, Aisy ZIR, Sutini T, et al.: Penggunaan media sosial dikalangan anak sekolah dasar. Harmony: Jurnal Pembelajaran IPS Dan PKN. 2022; 7(1): 48–52. Publisher Full Text Asafo-Adjei R, Mensah RO, Klu EK, et al.: Social media and English language writing performances of a Ghanaian ESL class: The nexus. Cogent Education. 2023; 10(1): 2189388. Publisher Full Text

Is the work clearly and accurately presented and does it cite the current literature?

Yes

If applicable, is the statistical analysis and its interpretation appropriate?

Yes

Are all the source data underlying the results available to ensure full reproducibility?

Yes

Is the study design appropriate and is the work technically sound?

Yes

Are the conclusions drawn adequately supported by the results?

Yes

Are sufficient details of methods and analysis provided to allow replication by others?

Yes

Reviewer Expertise:

The study is highly relevant to contemporary educational challenges and presents a clear, contextually grounded framework that integrates inclusivity, digital literacy, and language learning.

I confirm that I have read this submission and believe that I have an appropriate level of expertise to confirm that it is of an acceptable scientific standard.

# Reviewer report: Reviewer response for version 1

**DOI:** 10.5256/f1000research.189766.r446073

**Published:** 2026-01-14

**Authors:** Zulfin Rachma Mufidah

**Affiliations:** 1 Universitas Negeri Surabaya, Surabaya, East Java, Indonesia

Firstly,

 in the section of Data Analysis, the manuscript states that *“NVivo 14 was used to manage qualitative data and visualize coding structures,”* the visualization of the qualitative findings has not yet been explicitly presented. This section therefore provides a clearer explanation and representation of how NVivo was used to support the analytical process. Please visualize the data scientifically.

 Secondly,

 a number of the cited sources are relatively dated and may not adequately represent the current state of research. Journal article citations are limited to publications from the past five years.

 Clarke V, Braun V: Teaching thematic analysis: Overcoming challenges and developing strategies for effective learning. The Psychologist. 2013; 26(2): 120–123

 Valencia JAÁ: Language views on social networking sites for language learning: the case of Busuu. Comput. Assist. Lang. Learn. 2016; 29(5): 853–867

 Zhuravleva A, de Bot K , Hilton NH: Using social media to measure language use. J. Multiling. Multicult. Dev. 2016; 37(6): 601–614

 Thirdly,

 avoid direct translation from Indonesian into English, instead, employ scientifically rigorous and academically appropriate language consistent with international journal standards.

Is the work clearly and accurately presented and does it cite the current literature?

Partly

If applicable, is the statistical analysis and its interpretation appropriate?

No

Are all the source data underlying the results available to ensure full reproducibility?

Partly

Is the study design appropriate and is the work technically sound?

Yes

Are the conclusions drawn adequately supported by the results?

Partly

Are sufficient details of methods and analysis provided to allow replication by others?

Partly

Reviewer Expertise:

Inclusive education in primary school and language education.

I confirm that I have read this submission and believe that I have an appropriate level of expertise to confirm that it is of an acceptable scientific standard, however I have significant reservations, as outlined above.

# Response

**Published:** 2026-01-19

**Authors:** Megan Asri Humaira

This article has been updated based on reviewer feedback. Several sections have been updated, including:

 First, the presentation of images based on in vivo results;

 Second, the references used;

 Third, the previous grammar has used proofreading.

 Thank you for your input on this article.
